# Long Noncoding RNA PVT1 as a Novel Predictor of Metastasis, Clinicopathological Characteristics and Prognosis in Human Cancers: a Meta-Analysis

**DOI:** 10.1007/s12253-018-0451-3

**Published:** 2018-08-06

**Authors:** Congmin Liu, Jing Jin, Di Liang, Zhaoyu Gao, Yachen Zhang, Tiantian Guo, Yutong He

**Affiliations:** Cancer Institute, The Fourth Hospital of Hebei Medical University/The Tumor Hospital of Hebei Province, Shijiazhuang, 050000 Hebei China

**Keywords:** Long non-coding RNA, Plasmacytoma variant translocation1, Metastasis, Clinical stage, Prognosis, Meta-analysis

## Abstract

The present meta-analysis aimed to systematically evaluates the metastasis, clinical stage, and prognostic value regarding the expression levels of PVT1 in various cancers. Relevant literatures were searched in PubMed、Cochrane Library、Wed of science、Embase databases、Chinese National Knowledge Infrastructure and Wanfang from inception up to 22 August 2017. After data were extracted, a meta-analysis was performed using Review Manager 5.3 and Stata 12.0 software. The meta-analysis showed that high expression of PVT1 could predict more lymph node metastasis (LNM) (Odds ratio, OR = 2.83, 95% confidence interval, CI: 1.76–4.54, *P* < 0.0001), distant metastasis (DM) (OR = 3.60, 95% CI: 1.08–12.03, *P* = 0.04), advanced clinical stage (OR = 4.37, 95% CI: 3.45–5.54, *P* < 0.00001) and poor overall survival (Hazard ratio, HR = 2.08, 95% CI: 1.82–2.37, *P* < 0.00001)in cancer. Subgroup analysis in different systems also showed the same results, including respiratory system、digestive system、urinary system and other systems, especially in respiratory system (LNM, OR = 4.57, 95% CI: 2.41–8.68, *P* < 0.00001; clinical stage, OR = 5.59, 95% CI: 3.59–8.71, *P* < 0.00001; OS, HR = 2.43, 95% CI: 1.98–2.99, *P* < 0.00001). These results suggest that PVT1 could serve as a novel biomarker for metastasis, clinical stage and poor prognosis in various tumors.

## Introduction

With the incidence and mortality increased year by year, cancer is becoming a major public health problem and a leading cause of death worldwide. According to GLOBCAN 2012, there were 14.1 million new cancer cases and 8.2 million cancer deaths in 2012 worldwide [1]. In the United States, cancer is the second leading cause of death with an estimated 1,685,210 new cases and 595,690 deaths cancer in 2016 [2]. In China, cancer has been the leading cause of death with an estimated 4,292,000 new cases and 2,814,000 death cases in 2015 [3]. Lymph node metastasis, distant metastasis and clinical stage play an important role in the progression of cancer and are closely related to the prognosis of cancer. The presence of lymph node metastasis and distant metastasis also determines the treatment of cancer, such as surgery, radiotherapy or chemotherapy. Therefore, looking for molecular markers associated with metastasis, clinical stage and prognosis is becoming imminent for the therapy of cancer.

Long noncoding RNAs (lncRNAs) are a class of RNAs with a length greater than 200 nucleotides and no protein coding ability. Recently, with the rapid development of high throughput sequencing technology, lncRNAs have been found to be abnormally regulated in various types of cancer and played an indispensable role in the metastasis, advanced clinical stage and prognosis of cancer [4]. Plasmacytoma variant translocation1(PVT1) was first discovered in 2013 in human colorectal cancer and was a copy number amplification associated lncRNA which located on chromosome 8q24 and near MYC [5]. Accumulating evidence revealed that PVT1 was unregulated and played vital regulatory roles in a variety of cancers, including colorectal [6], pancreatic [7], breast and ovarian cancer [8]. High PVT1 expression was strongly correlated with clinicopathologic characteristics, such as metastasis, clinical stage and prognosis [9, 10]. However, since the results of the studies were not consistent (15 articles displayed positive results and 9 articles showed negative results) and small sample size in individual study, we collected relevant publications and performed a meta-analysis to investigate the relationship between PVT1 expression and metastasis, clinical stage or prognosis, aiming to further evaluate whether the PVT1 could be served as a potential molecular biomarker for cancers.

## Material and Methods

### Search Strategy and Literature Selection

We searched the electronic databases PubMed、Cochrane Library、Wed of science、Embase databases、Chinese National Knowledge Infrastructure and Wanfang, by using “PVT1 or plasmacytoma variant translocation1” as the keyword, in order to obtain potential articles referenced in the publications. Retrieval time for the last update is up to 22 August 2017.

### Inclusion and Exclusion Criteria

Inclusion criteria for the articles were as the following: (1) Case-control studies that evaluate the relationship between PVT1 expression and metastasis, clinical stage or prognosis of patients in human cancer. (2) Patients were divided into high and low expression group according to PVT1 expression. (3) Related clinicopathologic parameters were described, such as lymph node metastasis、distant metastasis and clinical stage. (4) Related outcomes were reported, including overall survival (OS)、disease-free survival (DFS)、progression free survival (PFS)、recurrence free survival (RFS). (5) Sufficient data for calculating OR、HR and its corresponding 95% confidence intervals (CI).

Exclusion criteria for the articles were as follows: (1) Nonhuman research, reviews, editorials, expert opinions, letters and case reports. (2) Duplicate publications. (3) Studies without valuable data.

### Date Extraction and Quality Assessment

Two investigators(YTH, CML)extracted and reviewed the essential data according to the inclusion and exclusion criteria independently. For each eligible study, we extracted the following information: first author, publication year, tumor type, country, total number of patients, detection method of PVT1 expression levels, number of high PVT1 expression group and low PVT1 expression group, number of patients with lymph node metastasis, distant metastasis and different clinical stage, follow-up duration, cut-off value, HRs as well as their 95% CIs. The quality of all eligible studies was assessed by two investigators (YCZ, ZYG) according to the Newcastle-Ottawa Scale independently. NOS scores ranged from 0 to 9 points, with higher scores indicated a better quality and all included eligible studies were assessed to be of high quality by using the NOS in this meta-analysis.

### Statistical Analysis

The association between PVT1 and cancer metastasis, clinical stage, or prognosis was assessed by OR and HR with its corresponding 95% CI. The current meta-analysis was performed through Review Manager 5.3 and Stata 12.0 software. We use the Chi square-based Q test and I^2^ statistics evaluate the heterogeneity of the eligible studies. The random-effects model was used to analyze the results when heterogeneity was present (*I*^*2*^ > 50%, *P* < 0.05); while the fixed-effects model was applied for this meta-analysis when the heterogeneity was absent (*I*^*2*^ < 50%, *P* > 0.05). The potential publication bias was assessed with the Begg’s funnel-plot. *P*-value less than 0.05 were considered to be statistically significant.

## Results

### Literature Search and Study Characteristics

**Fig 1:**
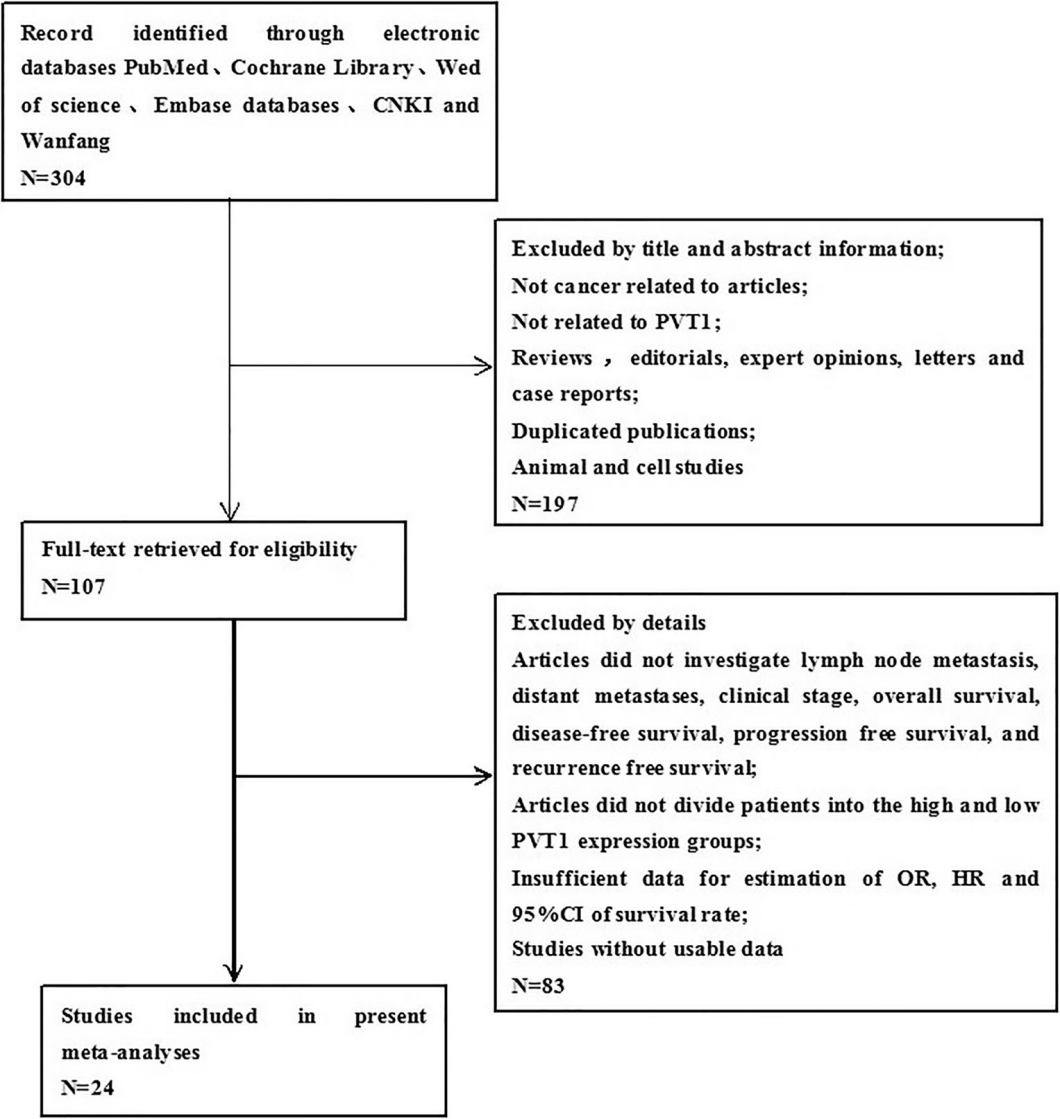
Flowchart of selecting studies for inclusion in this meta-analysis

According to the inclusion and exclusion criteria, a total of 24 eligible studies [9–32] were screened upon an electrical search (Fig. 1). These studies included a total of 2212 patients; and the patient^’^s sample size ranges from 28 to 214 with the mean value of 92. Among the 24 studies, 6 studies focused on gastric cancer, 4 on non-small cell lung cancer and hepatocellular cancer respectively, one on lung cancer, colorectal cancer, pancreatic cancer, pancreatic ductal adenocarcinoma, renal cancer, bladder cancer, esophageal cancer, cervical cancer, epithelial ovarian cancer, and osteosarcoma respectively. All the diagnoses of lymph node metastasis, distant metastasis and tumor–node–metastasis (TNM) were based on pathology. In all of the included studies, the patients were divided into two groups: high and low expression of PVT1. All studies used qRT-PCR to detect the expression of PVT1. The main characteristics of the eligible studies were summarized in Tables 1 and 2.

**Table 1:** Metastasis and clinical stage of the eligible studies in this meta-analysis

Author	Year	Tumor type	Country	Sample size	Detection method	cut-off value	PVT1 expression							
							High expression	High with LNM	High with DM	High with advanced clinical stage	Low expression	Low with LNM	Low with DM	Low with early clinical stage
Cui	2015	NSCLC	China	108	qRT-PCR	median value	53	29	2	28	55	19	1	13
HuangCS	2016	SCLC	China	120	qRT-PCR	median value	60	45	27	46	60	15	4	17
Wan	2016	NSCLC	China	105	qRT-PCR	median value	56	36	NA	48	49	19	NA	27
Wu	2017	NSCLC	China	31	qRT-PCR	median expression	15	9	NA	12	16	3	NA	6
Yang	2014	NSCLC	China	82	qRT-PCR	median value	65	37	NA	39	17	2	NA	3
DingJ	2014	GC	China	31	qRT-PCR	cancer/noncancerous tissue > 1.0	19	15	3	14	12	4	0	4
Gou	2017	HCC	China	92	qRT-PCR	NA	48	NA	NA	23	44	NA	NA	10
HuangC	2015	PDAC	China	85	qRT-PCR	mean value	67	28	11	46	18	13	4	7
Huang T	2017	GC	China	68	qRT-PCR	fold-change ≥/≤ mean ratio	30	NA	NA	21	38	NA	NA	16
Kong	2015	GC	China	80	qRT-PCR	median value	40	24	NA	26	40	18	NA	13
Lan	2017	HCC	China	48	qRT-PCR	median value	24	NA	NA	19	24	NA	NA	11
Ren	2016	GC	China	28	qRT-PCR	NA	13	11	1	10	15	6	0	9
Takahashi	2014	CRC	Japan	164	qRT-PCR	expression higher or lower than the 20 percentile value	131	69	3	74	33	9	1	9
Wang	2014	HCC	China	89	qRT-PCR	median value	45	18	NA	19	44	15	NA	9
Yuan	2016	GC	China	111	qRT-PCR	median value	55	30	2	29	56	19	1	14
Zhao	2017	PC	China	34	qRT-PCR	NA	18	NA	17	16	16	NA	3	5
Zheng	2016	EC	China	77	qRT-PCR	median value	39	23	NA	27	38	7	NA	15
HuangY	2015	RC	China	54	qRT-PCR	median value	39	16	NA	24	15	3	NA	4
Zhuang	2015	BC	China	32	qRT-PCR	NA	20	1	NA	19	12	2	NA	5
Song	2017	OC	China	46	qRT-PCR	mean value	24	NA	NA	16	22	NA	NA	3
Total				1485			861	391	66	556	624	154	14	200

NSCLC: non-small cell lung cancer, SCLC: small cell lung cancer, GC: gastric cancer, PDAC: pancreatic ductal adenocarcinoma, CRC: colorectal cancer, HCC: hepatocellular carcinoma, EC: esophageal cancer, RC: renal carcinoma, BC: bladder cancer, PC: pancreatic carcinoma, OC: osteosarcoma, qRT-PCR: quantitative real-time PCR, NA: not available

**Table 2:** Overall survival of the eligible studies in this meta-analysis

Study	Year	Disease	Country	Number	Detection method	Survival analysis	Multivariate analysis	HR statistic	Hazard ratios (95% CI)	Follow up (months)
Cui	2015	NSCLC	China	108	qRT-PCR	OS DFS	Yes	Data in paper	1.72 (1.14–3.25)	40
HuangCS	2016	SCLC	China	120	qRT-PCR	OS	Yes	Data in paper	1.782 (1.078–2.945)	96
Wan	2016	NSCLC	China	105	qRT-PCR	OS PFS	Yes	Data in paper	2.464 (1.214–4.999)	40
Wu	2017	NSCLC	China	31	qRT-PCR	OS	NO	Survival curve	3.19 (1.16–8.77)	80
Yang	2014	NSCLC	China	82	qRT-PCR	OS	Yes	Data in paper	3.273 (2.184–6.937)	60
DingC	2015	HCC	China	214	qRT-PCR	OS RFS	Yes	Data in paper	0.91 (0.59–1.41)	120
HuangC	2015	PDAC	China	85	qRT-PCR	OS	Yes	Data in paper	3.3013 (1.574–6.673)	60
Kong	2015	GC	China	80	qRT-PCR	OS DFS	Yes	Data in paper	2.092 (1.068–4.096)	40
Lan	2017	HCC	China	48	qRT-PCR	OS	NO	Survival curve	2.65 (0.89–7.92)	48
Takahashi	2014	CRC	Japan	164	qRT-PCR	OS	Yes	Data in paper	2.532 (1.152–10.747)	>120
Wang	2014	HCC	China	89	qRT-PCR	OS RFS	NO	Survival curve	1.77(0.69–4.50)	50
Yuan	2016	GC	China	111	qRT-PCR	OS PFS	Yes	Data in paper	2.280 (1.054–4.930)	40
Zhao	2017	PC	China	34	qRT-PCR	OS	NO	Survival curve	1.66 (0.57–4.84)	>14
Marissa	2015	Cervical	USA	121	qRT-PCR	OS	NO	Survival curve	1.84(0.88–3.84)	60
Paolo	2017	EOC	Italy	202	qRT-PCR	OS PFS	Yes	Data in paper	2.1 (1.4–3.3)	200
Song	2017	OC	China	46	qRT-PCR	OS	NO	Survival curve	1.79 (0.51–6.32)	72
Total				1640						

NSCLC: non-small cell lung cancer, SCLC: small cell lung cancer, GC: gastric cancer, PDAC: pancreatic ductal adenocarcinoma, CRC: colorectal cancer, HCC: hepatocellular carcinoma, PC: pancreatic carcinoma, OC: osteosarcoma, EOC: epithelial ovarian cancer, OS: overall survival, DFS: disease-free survival, PFS: progression free survival, RFS: recurrence free survival

## Meta-Analysis Results

### Association between PVT1 and LNM

**Fig. 2:**
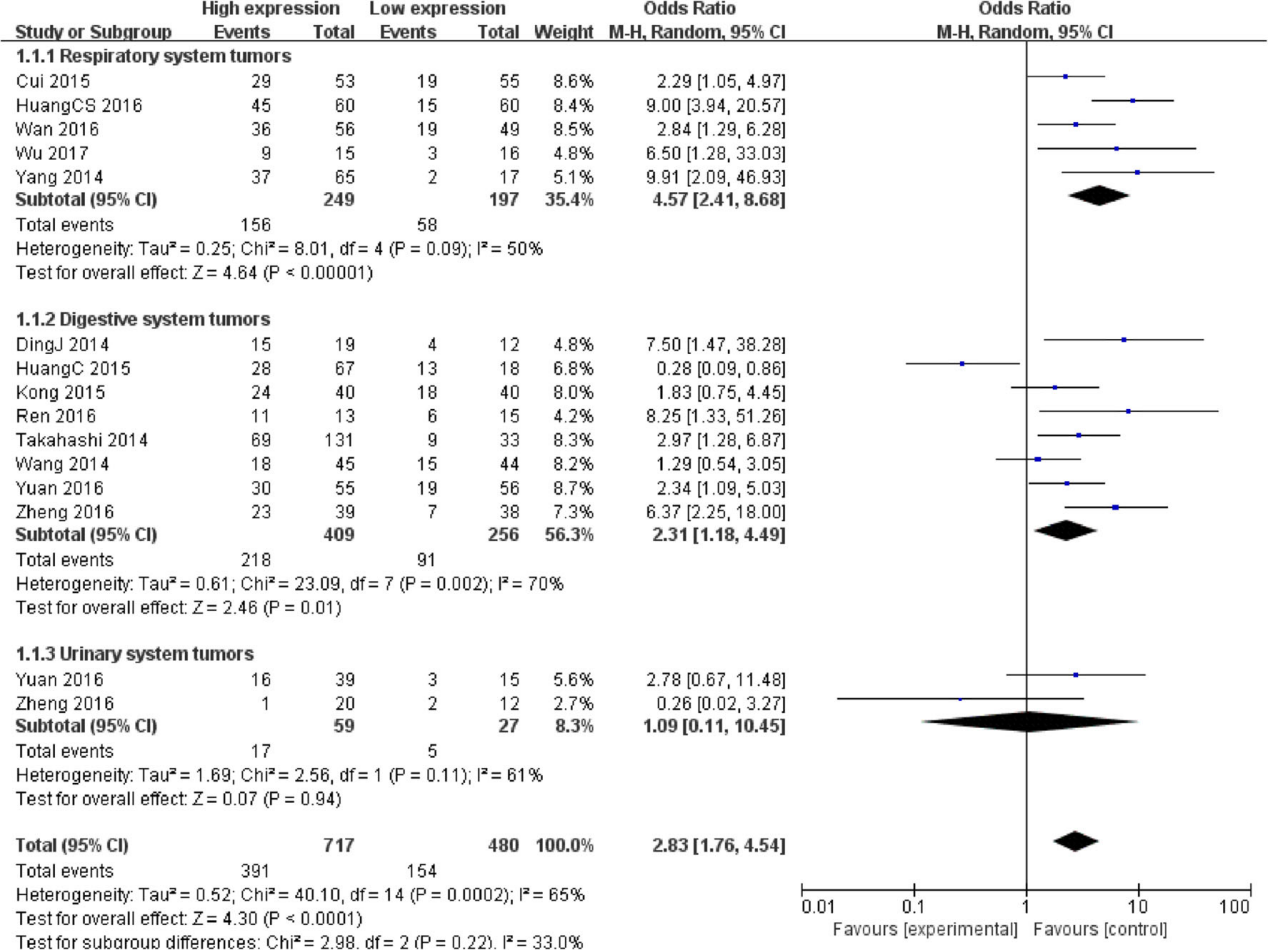
Forest plot for the association between PVT1 expression levels with LNM

15 studies reported 1197 patients with LNM based on different PVT1expression levels. The random-effects model was adopted as the significant heterogeneity (*I*^*2*^ = 65%, *P* = 0.0002). Analysis showed that the OR of high PVT1 expression group versus low PVT1 expression group was 2.83 (95% CI: 1.76–4.54, *P* < 0.0001) (Fig. 2), which revealed that a higher PVT1 expression predicted more LNM.

The subgroup analysis according to different systems in cancer types revealed a significant association between increased PVT1 expression and LNM in patients with respiratory system tumors (OR = 4.57, 95% CI: 2.41–8.68, *P* < 0.00001) and digestive system tumors (OR = 2.31, 95% CI: 1.18–4.49, *P* = 0.01). However, in urinary system tumors, the pooled result showed that cancer patients with high PVT1 expression were more likely to develop to LNM through no statistical significance was observed (OR = 1.09, 95% CI: 0.11–10.45, *P* = 0.94) (Fig. 2). This may be due to the too few literatures inclusion in the urinary system.

### Association between PVT1 and DM

**Fig. 3:**
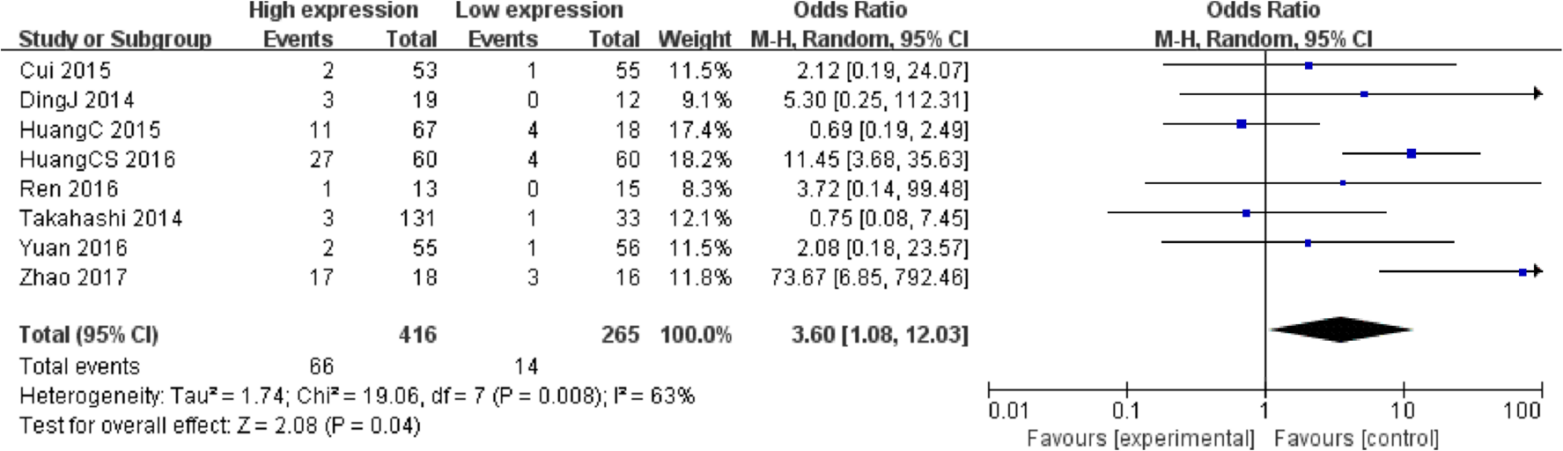
Forest plot of the correlation between PVT1 expression levels and DM in different cancer patients

681 patients were included in 8 studies assessed the association between PVT1 expression and DM. The random-effects model was applied as the significant heterogeneity (*I*^*2*^ = 63%, *P* = 0.008). Analysis showed that high PVT1 expression was more prone to DM (OR = 3.60, 95% CI: 1.08–12.03, *P* = 0.04) (Fig. 3).

### Association between PVT1 and Clinical Stage

**Fig. 4:**
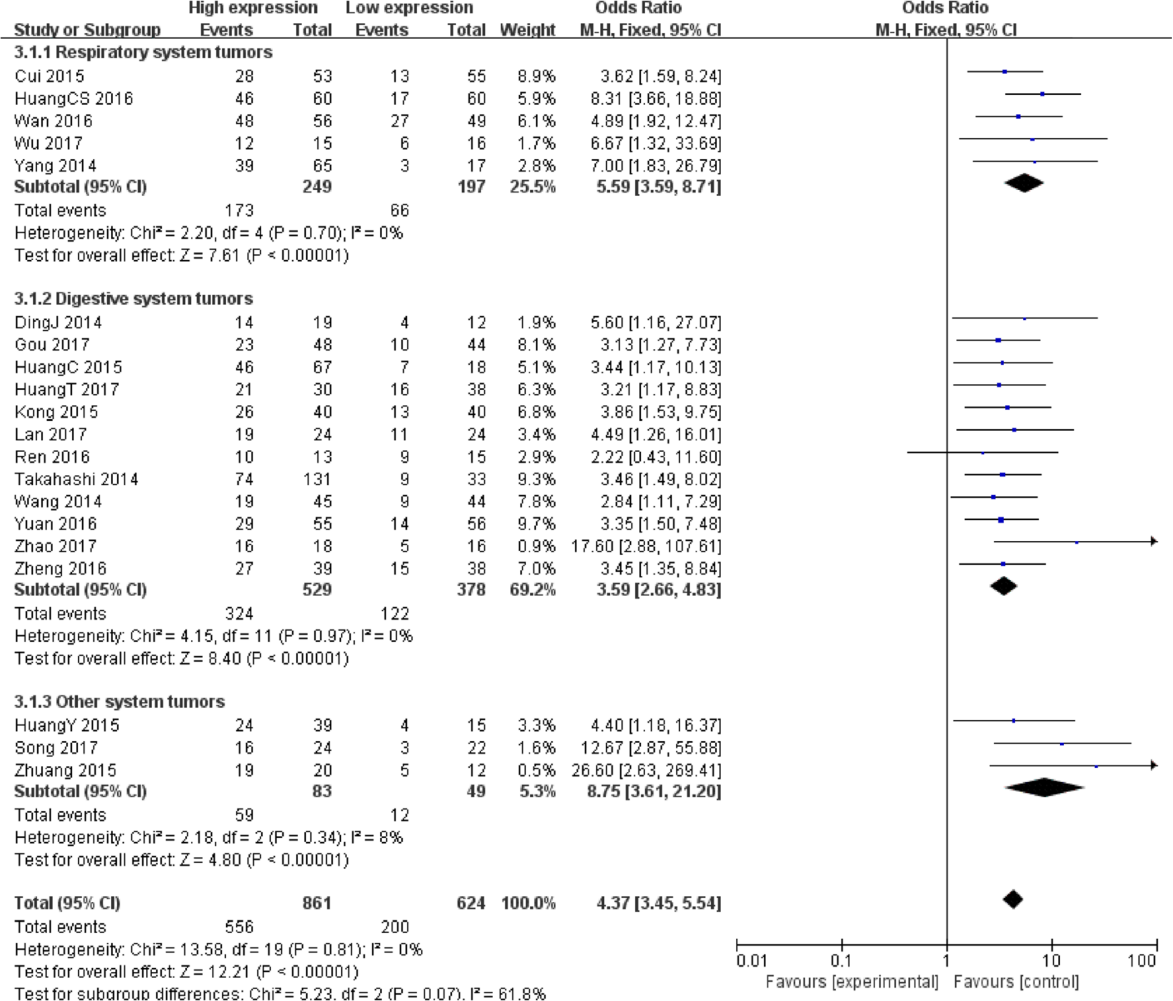
Forest plot for the association between PVT1 expression levels and clinical stage in different cancer patients

20 studies contain 1485 patients with clinical stage were included. The fix-effect model was used as no heterogeneity existed (*I*^*2*^ = 0%, *P* = 0.81). Analysis showed the OR of 4.37 with 95% CI: 3.45–5.54 (*P* < 0.00001) (Fig. 4), which revealed that a higher PVT1 expression was predictive of advanced clinical stage.

The subgroup analysis according to different systems in cancer types revealed a significant association between increased PVT1 expression and advanced clinical stage in patients with respiratory system tumors (OR = 5.59, 95% CI: 3.59–8.71, *P* < 0.00001), digestive system tumors (OR = 3.59, 95% CI: 2.66–4.83, *P* < 0.00001) and other system tumors (OR = 8.75, 95% CI: 3.61–21.20, *P* < 0.00001) (Fig. 4).

### Association between PVT1 and OS

**Fig. 5:**
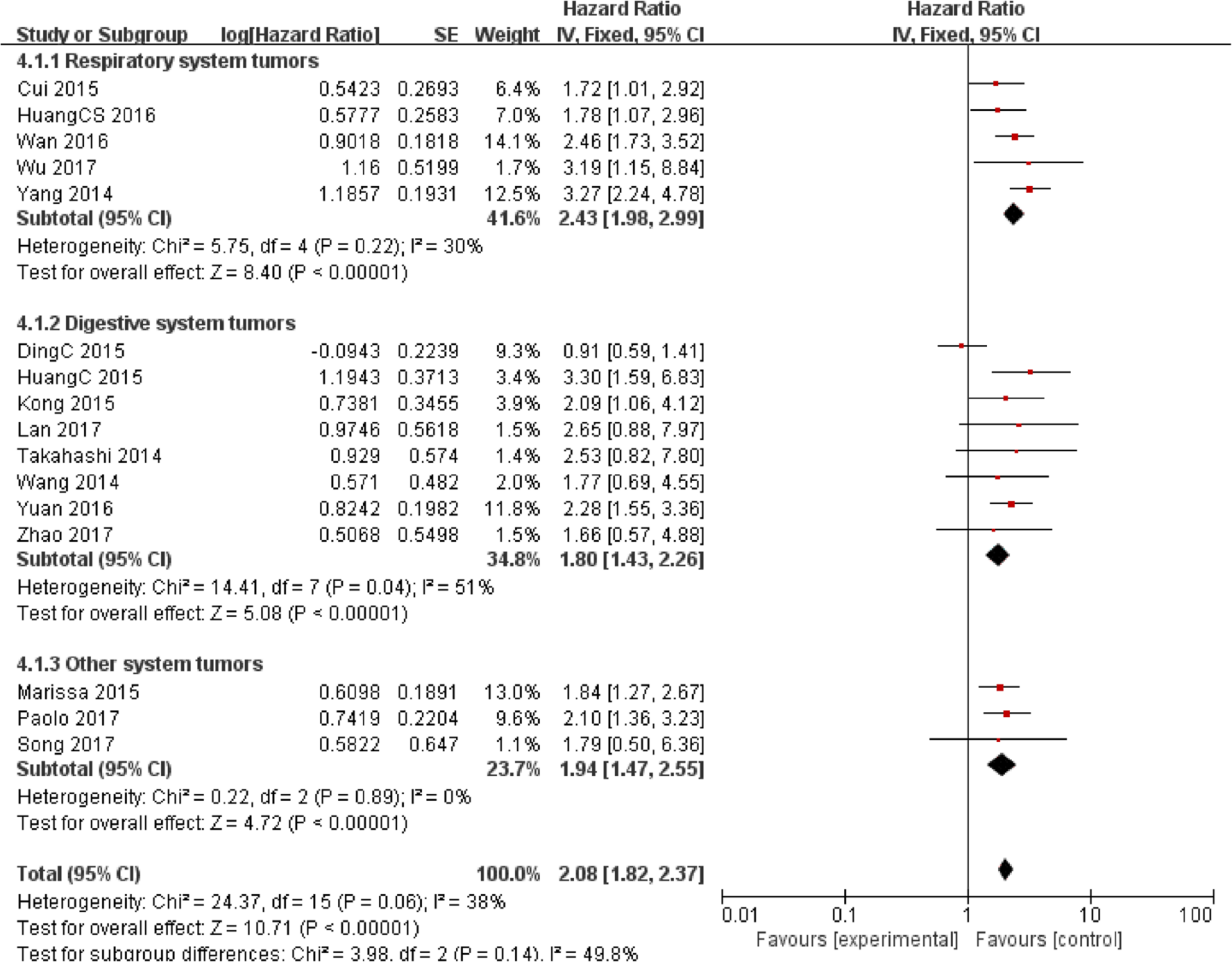
Forest plot for the association between PVT1 expression levels and OS for the included studies

16 studies reporting 1640 patients with OS were included according to different PVT1 expression levels. The fixed-effect model was used as the small heterogeneity existed (*I*^*2*^ = 38%, *P* = 0.06). Data of pooled HRs (HR = 2.08, 95% CI: 1.82–2.37, *P* < 0.00001) (Fig. 5) showed high PVT1 expression correlated with a worse survival.

The subgroup analysis also revealed a significant association between increased PVT1 expression and OS in patients with respiratory system tumors (HR = 2.43, 95% CI: 1.98–2.99, *P* < 0.00001), digestive system tumors (HR = 1.80, 95% CI: 1.43–2.26, *P* < 0.00001) and other system tumors (HR = 1.94, 95% CI: 1.47–2.55, *P* < 0.00001) (Fig. 5).

In additional, we investigated the association between PVT1 expression and DFS, PFS or RFS, respectively. The results revealed that significant negative association between PVT1 expression levels and DFS (HR = 1.87, 95% CI: 1.40–2.49, *P* < 0.0001), PFS (HR = 1.85, 95% CI: 1.47–2.32, *P* < 0.00001) or RFS (HR = 1.76, 95% CI: 1.19–2.61, *P* = 0.005) were existed. All the results were listed in the Table 3.

**Table 3:** Results of this meta-analysis about the association between PVT1 expression and DFS, PFS or RFS

Survival period	Eligible studies	Sample size	Heterogeneity (fixed)		Pool HR (95% CI)	Meta regression (*p* value)
			I^2^ (%)	p value		
Disease-free survival (DFS)	3	378	0	0.82	1.87 (1.40–2.49)	P < 0.0001
Progression free survival (PFS)	3	418	0	0.45	1.85 (1.47–2.32)	P < 0.00001
Recurrence free survival (RFS)	2	303	0	0.61	1.76 (1.19–2.61)	*P* = 0.005

### Publication Bias and Sensitivity Analysis

**Fig. 6:**
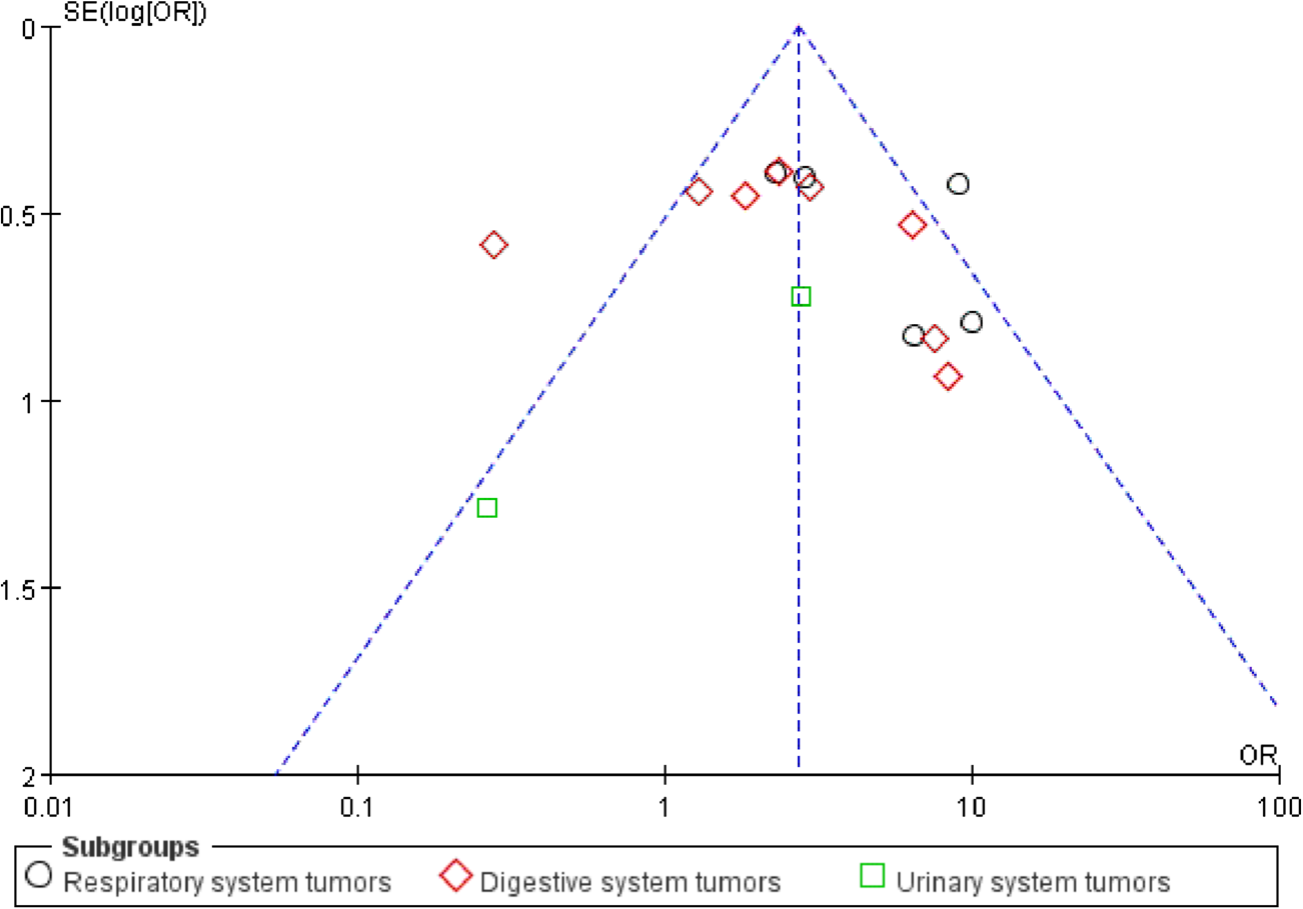
Funnel plot analysis of potential publication bias in LNM group

**Fig. 7:**
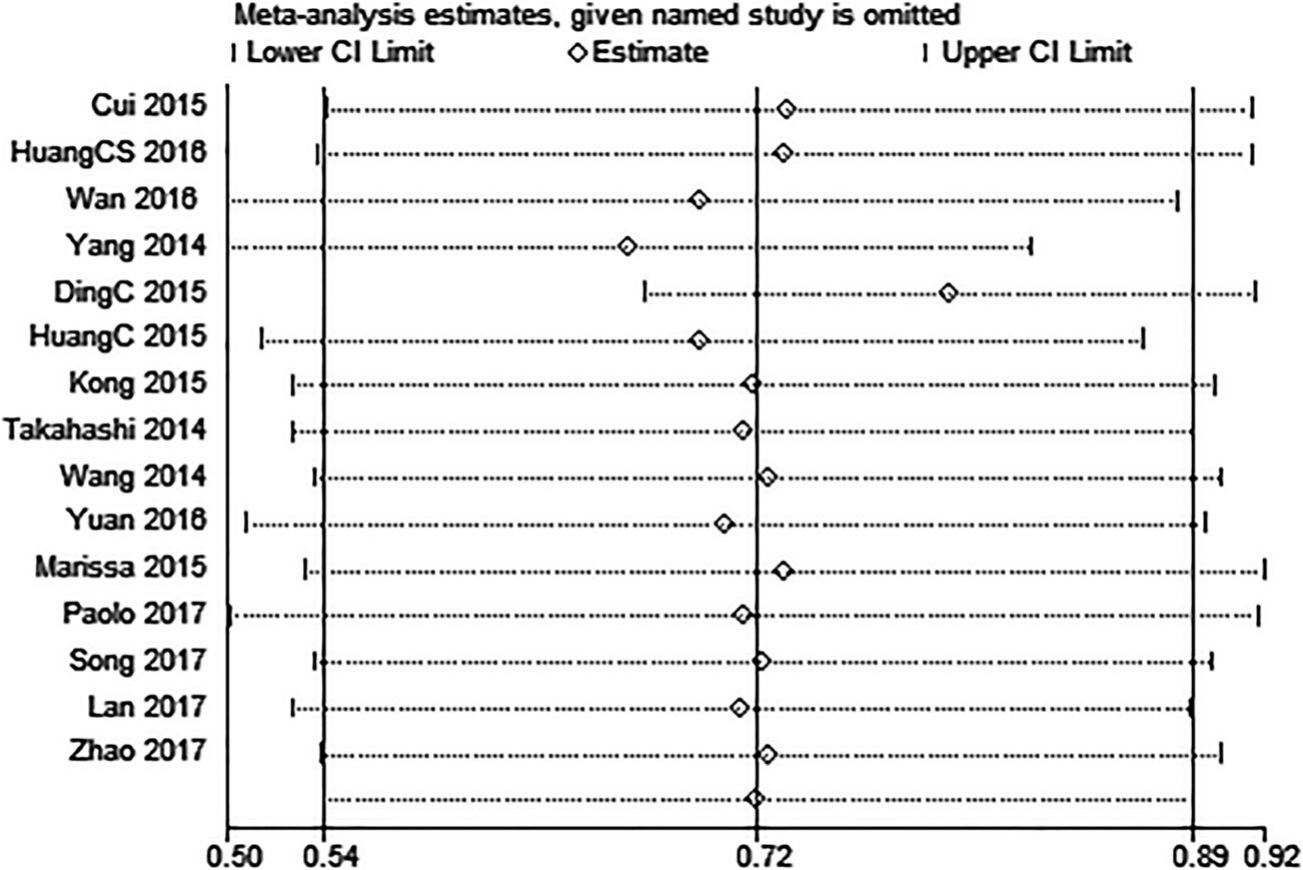
Sensitivity analyses of studies concerning PVT1 and overall survival

We use Egger’s test and funnel plot evaluate the publication bias of the present meta-analysis. Egger’s test (*P* = 0.728) revealed that there was no publication bias in analysis of LNM, and funnel plot (Fig. 6) showed no evidence of obvious asymmetry for LNM. Additionally,Similar results were also shown in the clinical stage and prognosis groups. Sensitivity analysis was carried out to evaluate the influence of a single study on the overall meta-analysis results by removing one study at a time in total population. When each study was omitted sequentially, the results were not significantly altered in this meta-analysis (Fig 7).

## Discussion

PVT1 was a novel lncRNA which was first discovered in 2013 in human colorectal cancer. In recent years, a growing number of studies have shown that PVT1 upregulated in several cancers. The further comprehensive mechanism between PVT1 and cancers was reported in continuance. PVT1 involved in tumor proliferation, invasion, migration and apoptosis and played an important role in tumor progression, metastasis and prognosis. In order to combine previous research results about PVT1 and cancers to arrive at a summary conclusion, we clarified the relationships between PVT1 expression levels and metastasis, clinical stage or prognosis in cancers in this meta-analysis. As far as we know, this is the first meta-analysis providing comprehensive insights into the correlation of PVT1 and cancer clinical stage. Results showed that the risk of lymph node metastasis and distant metastasis in high PVT1 expression group was 2.83 and 3.60 folds than those with low PVT1 expression group, respectively. The risk of developing into advanced clinical stage in PVT1 over expression patients was 4.37 times higher than those with low PVT1expression. PVT1 over expression patients with poor prognosis were 2.08 times lower in patients with low PVT1 expression. The same results were also shown in the subgroup analysis of different system tumors.

First, in respiratory system tumors, Wan et al. found that Over expression PVT1 inhibited the expression of LATS2 by binding to enhancer of zeste homolog 2 (EZH2) and promoted cell proliferation in non-small cell lung cancer [15]. Digestive system tumors such as in esophageal cancer, PVT1 upregulation decreased E-cadherinexpression and increased N-cadherin and vimentin expression, and induced epithelial-to-mesenchymal transition (EMT) process [12]. In gastric cancer, Xu et al. suggested that PVT1 facilitated gastric cancer cell proliferation and metastasis, and fulfilled its oncogenic functions in a FOXM1-mediated manner [26]. In hepatocellular carcinoma, Lan et al. demonstrated that PVT1 over expression was significantly correlated with vascular invasion, liver cirrhosis and TNM stage. Mechanism studies showed that PVT1 served as an endogenous sponge for miR-186-5p to reduce its inhibiting effect on yes-associated protein 1 and thus promoted the tumorigenesis of hepatocellular carcinoma [29]. In colorectal cancer, Takahashi et al. demonstrated high PVT1 expression exhibited greater lymph node metastasis, venous invasion and a poor OS compared with low PVT1 expression [21]. In pancreatic carcinoma, Zhao et al. found that PVT1 functions as an endogenous ‘sponge’ by competing for miR-448 binding to regulate the miRNA target SERBP1and therefore promotes the proliferation and migration of pancreatic carcinoma cells [28]. Furthermore, PVT1 expression levels were significantly correlated with metastasis and advanced clinical stage in urinary system tumors, such as bladder cancer and renal carcinoma. Huang et al. showed that high PTV1 expression was correlated with lymph node metastasis, advanced TNM stage and shorter OS. Knock down PVT1 decreased cell proliferation and enhanced cell apoptosis. Finally, PVT1 also functioned as critical regulator in the prognosis of other system tumors, including cervical carcinoma, ovarian cancer and osteosarcoma. Song et al. suggested that high PVT1 expression predicted poor prognosis. PVT1 over expression increased glucose uptake, lactate production, and the expression of HK2 in osteosarcoma cells. PVT1 could act as molecular sponge to repress the expression of miR-497 and promote the development of osteosarcoma [30]. 

To summarize, although mechanisms of PVT1 acted were not the same in various cancers, many similarities were still existed. First, PVT1 played a key role in cancers by interacting with miRNAs. An inverse association was observed between PVT1 and miR-186 expression levels which was observed in gastric cancer and hepatocellular cancer, while PVT1 functions as an endogenous ‘sponge’ by competing for miR-448 in pancreatic cancer. Second, PVT1 could also alter the expression of certain genes, including bind to EZH2 in non-small cell lung cancer and gastric cancer. PVT1 could interact with FOXM1 directly and increase its protein expression in gastric cancer and increased glucose uptake, lactate production, and the expression of HK2 in osteosarcoma cancer. Finally, PVT1 also affected cell cycle progression, such as promoted cell proliferation, migration and invasion and inhibited cell apoptosis in various cancers. In general, PVT1 expression was significantly associated with metastasis, clinical stage, and poor prognosis in various types of cancer in different systems.

Otherwise, as with other meta-analysis, it should be acknowledged that some limitations existed in this meta-analysis. First, the cutoff values of PVT1 high expression and low expression were lack of uniform standard in different types of cancer, which may result in some heterogeneity and affect the results of the study. Second, different studies have different postoperative regimens that may have a significant impact on OS, DFS, PFS and RFS. Third, since most studies report positive results and negative results are rarely published, the results of this study may overestimate the effect of PVT1 on cancers to a certain extent. Although there are some limitations, but this meta-analysis still has its noteworthy advantages. First, 24 literatures including a total of 2212 cases were included in this meta-analysis. The sample size included was the largest, which significantly improved the statistical efficiency and accuracy of the test. Second, the search databases were the most and cancer types were the most comprehensive in this meta-analysis compared with the previous reports. Third, lymph node metastasis and distant metastasis, OS、DFS、PFS and RFS were included in this study, which made the results more complete and comprehensive. Finally, the inclusion and exclusion criteria were more stringent and the quality of the literatures incorporated was higher.

In conclusion, despite the limitations described above, our meta-analysis reveals that upregulated PVT1 is significantly correlated with more metastasis, advanced clinical stage and poor prognosis in patients with various cancers. Furthermore, the significance of PVT1 in the metastasis, clinical stage and prognosis of respiratory system tumors is more obvious and can be used as a potential molecular marker to evaluate the prognosis of cancer. Nevertheless, the indication in the urinary system is relatively weak as the fewer samples; suggesting that we need to incorporate more studies in the urinary system tumors to validate this result.

## Abbreviations

*lncRNA*: long noncoding RNA
*PVT1*: plasmacytoma variant translocation1
*LNM*: lymph node metastasis
*DM*: distant metastasis
*OR*: odds ratio
*CI*: confidence interval
*HR*: hazard ratio
OS: overall survival
*DFS*: disease-free survival
*PFS*: progression free survival
*RFS*: recurrence free survival
*TNM*: tumor–node–metastasis
*CNKI*: Chinese National Knowledge Infrastructure
*NSCLC*: non-small cell lung cancer
*SCLC*: small cell lung cancer
*GC*: gastric cancer
*PDAC*: pancreatic ductal adenocarcinoma
*CRC*: colorectal cancer
*HCC*: hepatocellular carcinoma
*EC*: esophageal cancer
*RC*: renal carcinoma
*BC*: bladder cancer
*PC*: pancreatic carcinoma
*EOC*: epithelial ovarian cancer
*OC*: osteosarcoma
*qRT-PCR*: quantitative real-time PCR
*NA*: not available
*EZH2*: zeste homolog 2
*EMT*: epithelial-to-mesenchymal transition
